# Anorexia Nervosa Complicating Pediatric Crohn Disease—Case Report and Literature Review

**DOI:** 10.3389/fped.2018.00283

**Published:** 2018-10-09

**Authors:** Aedin Collins, Elizabeth Nolan, Michelle Hurley, Antoinette D'Alton, Séamus Hussey

**Affiliations:** ^1^National Centre for Paediatric Gastroenterology, Our Lady's Children's Hospital, Dublin, Ireland; ^2^Department of Child and Adolescent Psychiatry, Our Lady's Children's Hospital, Dublin, Ireland; ^3^Department of Paediatrics, Royal College of Surgeons of Ireland, Dublin, Ireland; ^4^Department of Paediatrics, University College Dublin, Ireland; ^5^National Children's Research Centre, Dublin, Ireland

**Keywords:** Crohn disease, adolescent, anorexia nervosa, BMI, weight loss

## Abstract

Crohn disease and anorexia nervosa share common symptoms of weight loss and reduced oral intake. The prevalence of both disorders has increased over time. Symptoms of Crohn disease and anorexia nervosa can mimic each other leading to a delayed diagnosis and requiring complex, multidisciplinary management. Here we present a case of a 15 year old girl with Crohn disease who subsequently developed anorexia nervosa, and review the published literature on the occurrence of both diagnoses.

## Introduction

Crohn disease (CD) is a chronic idiopathic inflammatory bowel disease characterized by transmural inflammation and granulomatous lesions in the bowel (1). Its prevalence is increasing worldwide (1). Presenting childhood features include anorexia, weight loss, diarrhea, abdominal pain, perianal disease, growth, and pubertal delay, with most children being diagnosed after age 10 years (1).

Anorexia nervosa (AN) is an eating disorder diagnosed by the following DSM V Criteria: restriction of energy intake relative to requirement leading to a significantly low body weight in the context of age, sex, developmental trajectory, and physical health; an intense fear of gaining weight or becoming fat, or persistent behavior that interferes with weight gain even though at a significantly low weight even though underweight; disturbance in the way one's body weight and shape is experienced, undue influence of body weight or shape on self- evaluation, or persistent lack of recognition of the seriousness of current low body weight (2). As per Swanson et al. (3), the lifetime prevalence of AN is 0.3% in both adolescent males and females and increasing in the developed world (3, 4). The prevalence of subthreshold AN- cases that appear similar but do not meet full diagnostic criteria- is 1.5% in females vs. 1% in males (3). The incidence of CD amongst adolescent males is 7.4 per 100,00 and 6 per 100,000 females per year. Benchimol et al. (5) Crohn disease should be considered in the differential diagnosis of AN, especially in younger patients (6, 7).

## Case

An 11 year old girl originally presented with a 3 month history of diarrhea, weight loss, perianal skin tags and a labial abscess. Her centiles for weight and height were < 3rd and 25th centile respectively. Her diagnostic endoscopy and biopsies revealed ileocolonic ulceration and granulomatous inflammation, consistent with CD.She responded well to exclusive enteral nutrition (EEN) induction treatment and had sustained remission on thiopurine therapy for 18 months. She then had a symptomatic relapse, including a 10 kg weight loss, and was commenced on infliximab, an anti-TNFα monoclonal antibody, which re-induced and maintained remission until age 15 years. Her weight had improved from the 3rd to the 25th centile. Infliximab dosage and infusion intervals were optimized according to therapeutic drug monitoring results. At clinical review aged 15 1/2 years, she reported excellent health without any symptoms.Notably, her weight was < 3rd centile, and lab results included raised inflammatory markers and hypoalbuminemia. There was deep ileocolonic ulceration on repeat endoscopy but neither fibrostenotic nor fistulating disease on radiologic imaging. The patient was admitted, adalimumab was commenced instead of infliximab and EEN was commenced at 2,400 kcal per day. By day 10 of admission her weight had continued to fall and her BMI was 12.4 kg/m2. Biochemical work up revealed a hyponatremic, hypokalemic metabolic alkalosis. Due to concerns of non-compliance with EEN, 24-h “one-on-one” supervision was commenced. Within days her weight increased. Following multidisciplinary team engagement, she divulged she was “terrified of being overweight,” “hated” when she was in remission and felt “uncomfortable” when her weight was over 45 kg. She had been restricting her food intake and “idolized thin women” according to her mother. Following an adolescent psychiatry assessment, a diagnosis of AN was established. She ultimately required short-term parenteral nutrition until her disease stabilized and adequate feeding was re-established. She was discharged after 2 months of intensive child psychiatry and psychology input and has remained stable as an outpatient.

## Discussion

Crohn disease and anorexia nervosa share certain indistinguishable clinical features at diagnosis. A number of published reports highlight delays in diagnosis of CD due to an initial presumptive diagnosis of anorexia nervosa (8–10). Guidelines from the American Psychiatric Association now advise consideration and exclusion of gastrointestinal disorders when reaching the diagnosis of anorexia nervosa (3).

The literature regarding delayed diagnosis of AN in patients with CD is limited. Just 6 case reports, 6 case series and 2 cohort studies (pediatric and adult) were eligible for inclusion in a recent systematic review of inflammatory bowel disease and eating disorders (6). This included patients with an initial diagnosis of either an eating disorder or inflammatory bowel disease. Anorexia nervosa was more commonly associated with CD than ulcerative colitis. Of a total of 17 patients with detailed information, 5 “used” inflammatory bowel disease activity to exacerbate their weight loss. Notwithstanding the limited patient numbers involved, the prognosis of this dual pathology appears grim. Five patients required intestinal resection, 2 developed toxic megacolon and 1 died from intestinal perforation. It is tempting to speculate how inflammatory bowel disease may be a risk factor for eating disorders, given the focus on diet, fear of abdominal pain, poor body image and poor emotional wellbeing, factors also noted in the patient in this report (7).

There are just 3 pediatric reports to date of anorexia nervosa complicating CD. The first case is of an 11 year old girl who developed AN following corticosteroid treatment of CD (11). Another patient presented with both AN and CD leading to a complex and delayed diagnosis (12). The third case had significant weight loss despite good control of her underlying CD (13). Management included admission to psychiatric units in 2 cases, and psychotherapy in the third (11–13). It is possible that changing body habitus during disease course may be a potential trigger factor for the development of AN.

Gastroenterologists should maintain an index of suspicion for development of AN in pediatric IBD patients. Anorexia nervosa is classically more prevalent in adolescent than pre- adolescent patients, and among females rather than males (3). Obsessional, conforming and perfectionistic personality traits are common amongst patients with AN (14, 15). Ongoing weight loss out of proportion with disease activity is a clinical “red flag,” especially among patients reporting satisfaction with current weight, normal dietary intake and good medication compliance (13). Reviewing weight and height centile charts at each clinic visit, regardless of reported clinical symptoms is essential. Concerns regarding changes to body image with treatment should also be anticipated and explored (10). Access to multidisciplinary team assessment of potential contributory biopsychosocial factors is essential for pediatric gastroenterologists. Factors including interpersonal relationships, bullying, family dynamics, and personality traits can influence the onset of AN (11, 12).

Inflammatory bowel diseases are associated with other mental health disorders (16). Eating disorders have also been associated with other auto-immune diseases (17). Rates of depression and anxiety in patients with CD are higher than in the reference population, even more than 5 years after initial diagnosis (16). Pediatric patients with inflammatory bowel disease have higher rates of depression, anxiety, and phobic disorders than either healthy controls or patients with other chronic pediatric illnesses (18). Multidisciplinary input and support for children and adolescents with inflammatory bowel disease is vital for elucidating such complex presentations and ongoing patient support.

Crohn disease is a complex and highly variable disease. The current case highlights the importance of considering eating disorders in patients with poor treatment responses, and of the challenge of discerning co-morbid AN in patients with significant inflammatory disease burden. Multidisciplinary input and support for children with CD disease is necessary, even while in remission. Concerns regarding non-compliance with treatments, abnormal eating habits or unexpected weight loss should prompt early multidisciplinary engagement.

## Ethics statement

Local requirements did not mandate Ethics Committee approval as this was a retrospective anonymised case report. Parental informed consent was obtained and permission given to include patient details.

## Author contributions

All authors listed have made a substantial, direct and intellectual contribution to the work, and approved it for publication.

### Conflict of interest statement

The authors declare that the research was conducted in the absence of any commercial or financial relationships that could be construed as a potential conflict of interest.
